# A machine learning algorithm to explore the drivers of carbon emissions in Chinese cities

**DOI:** 10.1038/s41598-024-75753-y

**Published:** 2024-10-09

**Authors:** Wenmei Yu, Lina Xia, Qiang Cao

**Affiliations:** School of Finance, Anhui University of Finance and Economics, Bengbu, 233030 China

**Keywords:** Carbon emissions, Machine learning, Extremely randomized trees, Partial dependence plots, City heterogeneity, Environmental impact, Environmental social sciences, Climate-change adaptation, Climate-change impacts, Climate-change mitigation, Climate-change policy, Energy and society, Environmental economics, Environmental impact, Socioeconomic scenarios, Sustainability

## Abstract

As the world’s largest energy consumer and carbon emitter, the task of carbon emission reduction is imminent. In order to realize the dual-carbon goal at an early date, it is necessary to study the key factors affecting China’s carbon emissions and their non-linear relationships. This paper compares the performance of six machine learning algorithms to that of traditional econometric models in predicting carbon emissions in China from 2011 to 2020 using panel data from 254 cities in China. Specifically, it analyzes the comparative importance of domestic economic, external economic, and policy uncertainty factors as well as the nonparametric relationship between these factors and carbon emissions based on the Extra-trees model. Results show that energy consumption (ENC) remains the root cause of increased carbon emissions among domestic economic factors, although government intervention (GOV) and digital finance (DIG) can significantly reduce it. Next, among the external economic and policy uncertainty factors, foreign direct investment (FDI) and economic policy uncertainty (EPU) are important factors influencing carbon emissions, and the partial dependence plots (PDPs) confirm the pollution haven hypothesis and also reveal the role of EPU in reducing carbon emissions. The heterogeneity of factors affecting carbon emissions is also analyzed under different city sizes, and it is found that ENC is a common driving factor in cities of different sizes, but there are some differences. Finally, appropriate policy recommendations are proposed by us to help China move rapidly towards a green and sustainable development path.

**Supplementary Information:**

The online version contains supplementary material available at 10.1038/s41598-024-75753-y.

## Introduction

Greenhouse gas emissions, the curse of industrialization, have created a severe threat to the global ecosystem. The sixth report of the Intergovernmental Panel on Climate Change (IPCC) for 2021 stated that greenhouse gas emissions over the past decade have reached the highest level in human history^1^. It emphasized that there would be little prospect of limiting global warming to 1.5 °C without immediate and drastic measures taken on emissions reductions^2^. This also put a tremendous strain on China, which has surpassed the U.S. in carbon emissions since 2004. In 2021 alone, China has emitted 11.47 billion tons of greenhouse gas, or almost 30.9% of the total emissions worldwide. Therefore, how to optimize its carbon emissions has constantly attracted the attention of the academic circle worldwide. China’s carbon emission reduction pathway is urgent for the early attainment of the dual-carbon target.

The STIRPAT model^3^ was used as a guide for early studies of the topic. With strict assumptions and derivations of this theoretical model, they established the dominant role of demographic, economic, and technological factors in the forecast of carbon emissions^4^. Recent research has uncovered a complex interplay, revealing an increasing number of positively related determinants. These include domestic economic factors^5^; external economic factors^6^ and uncertainties^7–9^, to name a few. Although these studies provide a new perspective into the issue, they usually employ variables from one single category to ensure the robustness of their estimates^10^;^11^. As a result, an integrated perspective is currently lacking to assess the relative importance and interrelationships of these determinants.

In light of this, we implemented machine learning techniques. These approaches are superior to traditional econometrics in many aspects. First, since there are no a priori requirements on the distribution, machine learning techniques allow flexibility in choosing the model’s functional form^12^. Second, we perform feature selection on multidimensional data to filter irrelevant variables, thereby avoiding the overfitting problem that plagues traditional econometrics^13^. proposed an adaptive-Lasso method, which incorporates weights to automatically adjust the penalty factors. Third, machine learning allows for interpretable prediction analysis because it ranks the importance of variables and analyzes partial dependency plots by evaluating the magnitude of each feature’s contribution, leaving policymakers with room to weigh the results. For instance^14^, identified the drivers of carbon intensity and predicted the trend in China using a random forest algorithm.

To fill the existing research gaps, we collected annual data of 254 cities in China from 2011 to 2020 from the China Statistical Yearbook. We used multiple machine learning algorithms for a comparative study and found that the Extra-Trees (Extremely Randomized Trees) algorithm is the most effective in studying the influencing factors of carbon emissions. It is not only an integrated learning method, but it also improves the model’s generalization ability and stability by constructing multiple extremely randomized decision trees.

There are three contributions to this paper. First, there are numerous factors influencing China’s carbon emissions. To prevent model overfitting, existing studies either focus on the effects of fewer factors or utilize the lasso model^13^, which fails to achieve the goal of screening variables. According to^15^, this paper employs the adaptive lasso model, which can eliminate factors that aren’t very important and make the prediction model simpler. This fixes the model’s overfitting problem and keeps it from losing its ability to generalize. Second, we suggest using several machine learning models to figure out what causes China’s carbon emissions. These algorithms will also show any non-parametric connections between carbon emissions and factors in China’s economy, factors in other economies, and policy uncertainty by making the output simple to understand. Not only does the method accurately guess China’s carbon emissions, it also looks at how each variable affects carbon emissions and how important they are by using a variable importance ranking and a partial correlation dependency plot^16^. Third, we also study the heterogeneity of drivers by city size, which aids in formulating more effective decisions to reduce urban carbon emissions. This will help China’s urban carbon emissions move towards a more sustainable path.

We structure the rest of the paper as follows: Part 2 is a literature review. Part 3 describes the data and theoretical methodology. Part 4 reports the results of the empirical study. Part 5 presents the conclusions and policy recommendations.

## Literature review

For the influencing factors affecting China’s carbon emissions, researchers have long identified the impact of a variety of domestic economic factors, such as urbanization rate^17^, degree of marketization^18^, industrial structure^19,20^, human capital^21^, financial development^22^, government intervention^23^, economic development^24,25^, energy consumption^26,27^, environmental regulation^28^, green total factor productivity^29^, green technological innovation^30,31^, digital finance^32^, and fintech^33^, Environmental, Social, and Governance (ESG) investments^34^, Economic complexity^35^. ^36^ and ^37^ argued that heavy use of conventional energy sources leads to excessive carbon emissions. Researchers also find that external shocks, including external openness^38^ and FDI^39^, may also affect China’s carbon emissions,. Finally, since pandemic, climatic and economic shocks exacerbate world uncertainty, economic policy uncertainty (EPU)^40^, pandemic uncertainty (WPU)^41^, and climate policy uncertainty (CPU)^42^ may also have a part in affecting carbon emissions.

But if all the above-mentioned influencing factors are added to the prediction model, overfitting emerges and reduces the external validity and generalizability of the estimates. Therefore, choosing an appropriate feature selection method is essential. Two types of dimension reduction methods, namely, unsupervised and supervised methods, are usually adopted. Unsupervised dimension reduction algorithms include principal component methods, relief algorithms, etc. For example^43^, used PCA to reduce the input data dimensionality by removing four principal constituents from the 16 potential influencing factors^44^. utilized an algorithm that combined the PACF and relief to filter the input variables. The supervised downscaling algorithm includes the lasso model, by which^45^ remove extraneous factors affecting the carbon price. ^15^ and ^46^ optimized on this basis by adjusting the penalty factor using the automatic introduction of weights, an approach known as the adaptive lasso model^13^.

Likewise, Scholars have proposed various prediction models to study the future trend of carbon emissions in China. Given the intent, these models are generally subdivided into two categories. One is referred to as unsupervised forecasting methods that predict based on the data. For example, scholars have used multi-stage clustering to predict carbon emissions in G20 countries^47^, and factor analysis to identify key variables and use ELM to predict carbon intensity^48^. However, without labeling the input data, the prediction results may be biased. The other is supervised learning with “labeling” which is more advantageous in prediction^49^. For instance, using decision trees to predict differences in carbon emissions across industries^50^. Other researchers have used integrated learning based on decision trees to improve the generalizability of individual base learners. For example, Xgboost, Random Forest and Bagging were used to predict carbon emissions^51^. Extra-trees, an algorithm optimized for Random Forests, have also been used to predict emissions trends in China’s steel industry^52^.

To sum up, the existing literature has the following insufficiencies. First, the current studies mainly use traditional econometric methods to study the relationship between carbon emissions and one or two independent variables, which avoids overfitting but fails to consider the influence of other equally important drivers. Second, machine learning models are often used to make predictions and few to interpret the results, not to mention discussing variable importance or conducting partial dependency plots. Third, the importance of factors influencing carbon emissions may vary as cities scale up, which makes an analysis of urban heterogeneity essential to the current research.

## Data description and theoretical methods

### Data description

This research sample covers annual data from 2011 to 2020 for 254 cities in China. We select from the existing literature and identify the drivers of carbon emissions. A total of 14 domestic economic factors, 2 external economic factors and 3 uncertainty factors, are selected and reported in Table 1. For data processing, a few outliers are treated as missing values, which are processed by linear interpolation and moving average to make up the values, and the data have been standardized. Sources of data are China Statistical Yearbook of Urban Construction, China Statistical Yearbook of Energy and China Statistical Yearbook, it can be seen from the descriptive statistics in Table 1. We found that the standard deviation of green technology and digital finance is large, 1452.45 and 68.43 respectively, which may contain outliers. For detailed information and descriptive statistics of this data, please refer to Appendix 1(Supplementary Table 1) in the Supplementary Information.

**Table 1:** Description of the data.

Group	Variable Title	Acronym	Mean	Std. dev.	Min	Max
Dependent Variable	Carbon emissions	CE	0.0939	0.1476	0.0024	1.3808
Domestic economic factors	Urbanization rate	URL	0.5608	0.1437	0.2232	1.1015
	Marketability	MKD	12.0036	2.2796	4.9596	19.6944
	Industrial structure	UIS	6.5373	0.3458	5.5436	7.6516
	Human capital levels	HCL	0.0187	0.0208	0.0001	0.1276
	Financial development	FIN	2.7977	1.7191	0.5879	21.3015
	Government intervention	GOV	0.1893	0.0822	0.0202	0.6775
	Economic levels	AGDP	5.4298	3.0862	0.8877	20.3489
	Energy consumption	ENC	0.0235	0.0369	0.0004	0.4067
	Market-inspired environmental regulation	MAR	0.0033	0.0015	0.0002	0.0124
	Command-based environmental regulation	COR	0.0755	0.0777	0.0000	0.7320
	Green Total Factor Productivity	GTFP	1.0020	0.0946	0.0022	1.8918
	Green Technology	GTEC	465.8663	1452.4520	0.0000	24,051
	Digital finance	DIG	176.5402	68.4331	21.2600	334.4781
	Fintech Development	FTE	3.2708	1.3337	0.6931	7.1277
External economic factors	Opening-up	OPEN	0.1819	0.2813	0.0004	2.3735
	Foreign Direct Investment	FDI	1.0258	2.2542	0.0001	25.9800
Uncertainty factors	Economic policy uncertainty	EPU	191.1382	107.6520	92.1142	390.3880
	World pandemic uncertainty	WPU	1.8080	5.1429	0.0000	17.2300
	Climate policy uncertainty	CPU	241.6926	222.9319	44.2845	687.6163

Source: our elaborations.

### Combined prediction model

**Fig. 1:**
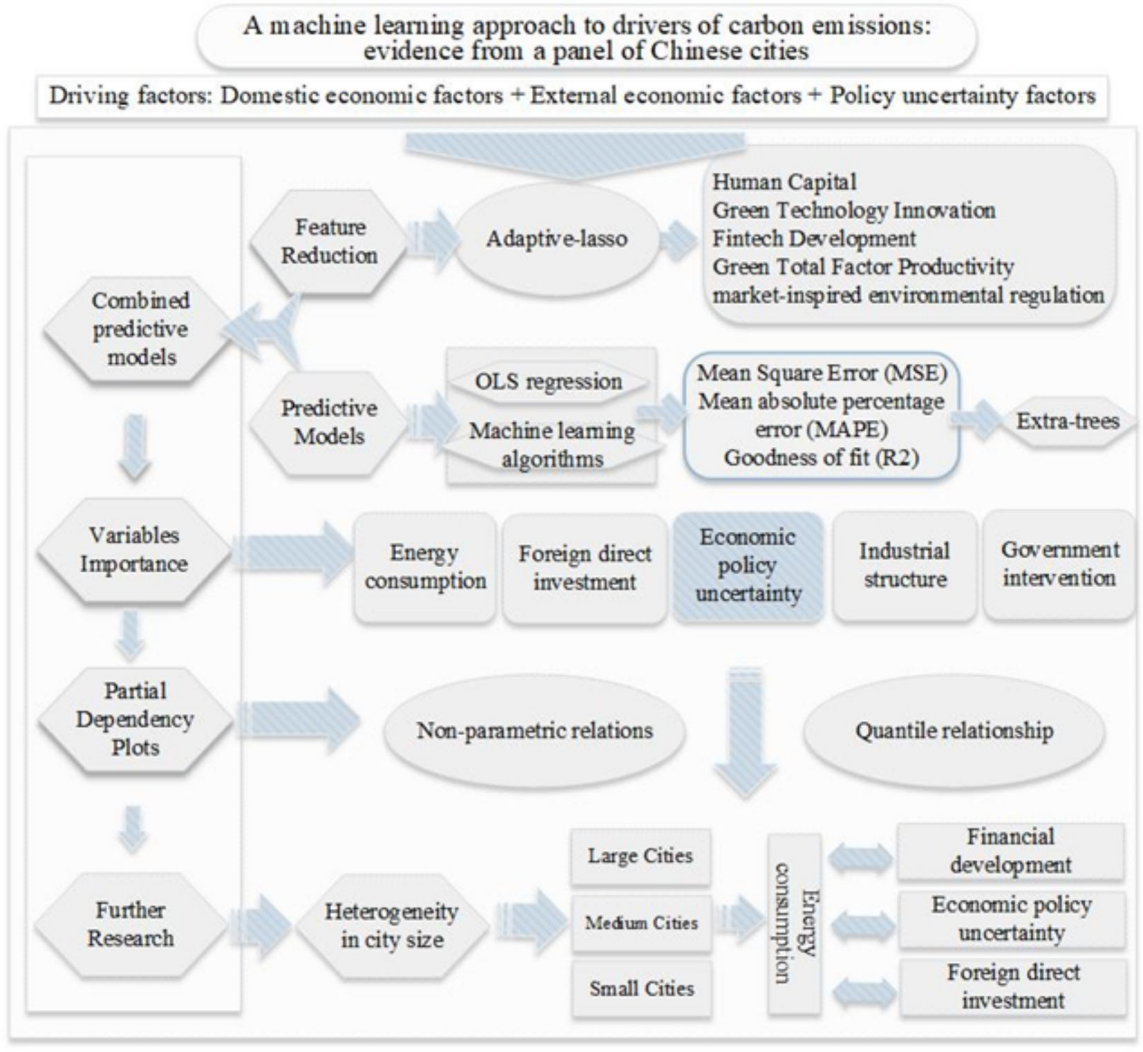
Research framework.

A framework for the research is shown in Fig. 1. While the literature review section explores the many factors that existing scholars believe influence China’s carbon emissions, from internal, external and uncertainty factors. First, based on the adaptive lasso model, the 19 influencing factors are screened, and those with low relevance to China’s carbon emissions are eliminated. Second, prediction studies are conducted on the feature-selected dataset through a variety of machine learning models. MSE, MAPE and R2 are used to assess the out-of-sample predictive power. Third, the importance of internal, external and uncertainty factors affecting China’s carbon emissions are ranked and assessed according to the training results of the machine learning model. Fourth, through the partial correlation dependency diagram, the predicted results are analyzed in an interpretable way, indicating the direction and magnitude. Fifth, the heterogeneity of the influencing factors is explored at different city sizes.

#### Feature selection

The Lasso model adds penalty terms to the generalized linear model to realize the feature selection function. And the Adaptive-Lasso model is able to use different penalty weights, which can better avoid model overfitting and improve the interpretability of the model, as detailed in Eq. (1):

1 $${\hat {\beta }_{{\text{alasso}}}}=\arg \hbox{min} (\parallel Y - X\beta {\parallel ^2}+\lambda \sum\limits_{{j=1}}^{p} {{{\hat {w}}_j}} \left| {{\beta _j}} \right|)$$

Where$${\hat {w}_j}=1/{\left| {\hat {\beta }} \right|^\gamma }$$$$(\gamma \succ 0)$$is the weighting function, $$\lambda$$ is the penalty parameter. The crucial for the Adaptive-Lasso model to implement variable selection is to determine a suitable weight function$${\hat {w}_j}$$, and the weights are obtained using cross-validation. The data used in this experiment was normalized based on the original data and the feature selection process for adaptive lasso was implemented using R software.

#### Multiple machine learning forecasting models

This paper selects Ordinary Least Square (OLS) and six multiple algorithms based on decision tree optimization to study the drivers of carbon emissions. They are OLS, Regression Tree (RT), Bagging, Random Forest (RF), Boosting, Xgboost, and Extremely randomized trees (Extra-trees). The traditional OLS regression and six machine learning algorithms are described in detail in Appendix 2. Where, OLS, Regression tree, Bagging models are implemented through R software. Random forest, Boosting, XGBoost and Extra-trees models are implemented through Python software.

According to^53^, all six machine learning models are based on decision tree algorithms with integrated learning. They improve the recognition rate and generalization of a single base learner by learning to obtain a decision tree base learner that combines the predictions of all base learners. Their advantage lies in their ability to achieve predictive interpretability in terms of variable importance and biased dependency graphs, which can better analyze the interrelationships. Due to space constraints, in addition to the Extra-trees model, the five machine learning models are described in detail in the Appendix 2, this paper focuses on the principle of the Extra-trees model.

The main steps of Extra-trees (Extreme Randomized Trees) are as follows:

Step 1: Input data set $D=\left\{ {({x_1},{y_1}),({x_2},{y_2}), \cdots ,({x_m},{y_m})} \right\}$, weak classifier iterations T and randomly selected samples from the training data;

Step 2: Feature selection. For $t=1,2, \cdots ,T$, at each node, a portion of the features are randomly selected;

Step 3: Split point selection. For each selected feature, a split point is randomly selected;

Step 4: Node splitting. Split nodes using randomly selected features and split points to create child nodes;

Step 5: Tree construction. Repeat the above steps until a predefined stopping condition is reached;

Step 6: Integration of results. The prediction results of all trees are integrated according to Eq. (2), and the final model output is the arithmetic mean of the T estimated coefficients:

2 $$\widehat {y}=\frac{1}{n}\sum\limits_{{i=1}}^{n} {(predictions)}$$

The model is very similar to the random forest model in that it consists of many decision trees. The difference is that Extra-trees select a random threshold to split the nodes. This approach not only reduces the high variance problem of RF, but also has a fast-performing speed. When training the model, Extra-trees select the entire dataset instead of using bootstrap samples. As a result, Extra-trees has better performance in the field of energy prediction compared to other integrated models^54^. The MSE is used as the Gini coefficient of the split values of the different features. Eventually, the generated n decision trees form Extra-trees, and the model predictions are the arithmetic mean of the estimated coefficients for each T.

This research used six machine learning models, of which 70% were training sets and 30% were test sets. In addition, the use of hyper-parameter tuning for a certain model leads to its prediction results being better than other models, the parameters of all models are set to the ideal values when the learning rate is 0.1, and no further parameters are specifically tuned. For example, the parameters of the Extra-trees model are: n_estimators = 300 are chosen for the experiments in this paper; max_features = n_features = 14 in the regression problem in this paper; and 10-fold cross-validation is chosen.

#### Predictive evaluation indicators

This research uses three validated predictors, MSE, MAPE and ${R^2}$, to evaluate the predictive performance of traditional OLS and six machine learning algorithms, which are calculated as detailed in Eqs. (3), (4), and (5). These three methods of predictive evaluation were implemented using Python software.

3 $$MSE=\frac{1}{n}\sum\limits_{{i=1}}^{n} {{{({y_i} - \hat {f}({x_i}))}^2}}$$

4 $$MAPE=\frac{1}{{\text{n}}}\sum\limits_{{i=1}}^{n} {\left| {\frac{{({y_i} - \hat {f}({x_i}))}}{{\hat {f}({x_i})}}} \right|} *100\%$$

5 $${R^2}=1 - \frac{{\sum\limits_{{i=1}}^{n} {{{({y_i} - \hat {f}({x_i}))}^2}} }}{{\sum\limits_{{i=1}}^{n} {{{({y_i} - \bar {f}({x_i}))}^2}} }}$$

where n is the number of samples, ${y_i}$ the actual value of CE, $\hat {f}({x_i})$the predicted value of CE, and $\bar {f}({x_i})$ the mean value of CE. Higher values of MSE and MAPE indicate higher prediction errors, and higher ${R^2}$ indicates better model fitting.

### Variable importance and partial dependency plots analysis

#### Variable importance

Feature importance refers to ranking of the importance of each feature in predicting carbon emissions^55^. It is implemented through Python software. In an ensemble containing K decision trees, ${x_j}$ is calculated as:

6 $$Imp({x_j})=\frac{1}{K}\sum\limits_{{k=1}}^{K} {\sum\limits_{{z \in {\varphi _k}}} {I({j_z}=j)[\frac{{{n_z}}}{N}\Delta i(z,s)]} }$$

where z denotes the zth non-terminal node of the decision tree ${\varphi _k}$, ${j_z}$the feature identifier used to split node z, $I()$the indicator function, ${n_z}$ the number of samples arriving at node z, N the total number of samples, and $\Delta i(z,s)$the amount of Gini coefficient that decreases at the zth node after s-splitting.

#### PDPs

To visualize the prediction results, the PDPs are used to plot the marginal effects of each influencing factor on China’s carbon emissions. It is implemented through Python software. Since $f( \cdot )$ has no analytic expression, it is generally difficult to compute this expectation directly. Therefore, replacing the overall mean ${E_{{x_2}, \cdots {x_p}}}( \cdot )$ with the sample mean can be obtained:

7 $$\hat {\phi }({x_1}) \equiv \frac{1}{n}\sum\limits_{{i=1}}^{n} {f({x_1},{x_i}_{2}, \cdots ,{x_{ip}})}$$

Arbitrarily given ${x_1}$, $\hat {\phi }({x_1})$ can be computed and the visualization of $({x_1},\hat {\phi }({x_1}))$drawn as partial dependency plots.

### Steps of the empirical study

**Table 2:** Steps of the empirical study. A total of eight steps are included here, which can be seen in table 2.

Steps:
Data collection and processing:
1: Identify the drivers.
2: Data collection and standardization of all variables.
**Combined prediction model: (R software**,** Python)**
3: Build the Adaptive-Lasso model according to Eq. (1) and complete the feature selection.
4: The drivers after feature selection are incorporated into OLS and six machine learning models for prediction, respectively.
5: The performance of the seven prediction models is evaluated according to Eqs. (3), (4), and (5).
**variable importance plots**:
6: The decrease in Gini coefficient is calculated and ranked according to Eq. (6).
**Partial Dependence Plots**:
7: Analyze PDPs according to Eq. (7), observe the direction and magnitude of each driver’s influence on carbon emissions, and study the nonparametric and quantile relationships between them.
**A study of heterogeneity in city size**:
8: The sample of 254 cities is classified according to city size to study the heterogeneity in the importance of variables.

## Results of the empirical research

### Feature selection

To avoid overfitting, the adaptive Lasso model is used to screen the 19 drivers initially and shrink the irrelevant factors and the results are shown in Table 3. The estimated coefficients of HCL, MAR, GTFP, GTEC and FTE on CE are 0, which indicates that these variables are not key influences on carbon emissions. From the remaining 14 variables affecting carbon emissions, we find that ENC is the variable that most affects carbon emissions, which is also uniformly considered by all scholars. In addition to this, we note that the uncertainty factor also seems to be an important variable that affects carbon emissions and that this influence is even higher than most of the internal and external economic factors^56^. also concluded that uncertainty is a major factor in the increase ofCE.

**Table 3:** Feature selection estimation results.

Variables	URL	MKD	UIS	HCL	FIN	GOV	AGDP	ENC	MAR	COR
CE	0.028	-0.006	0.074	0.000	-0.005	0.004	-0.150	0.813	0.000	0.005
Variables	GTFP	GTEC	DIG	FTE	OPEN	FDI	EPU	WPU	CPU	
CE	0.000	0.000	-0.030	0.000	0.016	0.149	-0.147	0.011	0.063	

Source: our elaborations.

Notes: This feature selection process is based on the overall panel data.

### Comparison of predicted results

The adaptive lasso model can only perform the feature selection, but it is difficult to determine the direction of the influence of each factor. Therefore, Regression tree, Bagging, Random Forest, GBM, XGBoost and extra-trees, are used in this paper for this purpose. Before the research, it is determining the multivariate prediction model for carbon emissions through three evaluation indexes: MSE, MAPE and R^2^. According to the results of Table 3 adaptive lasso shrinkage of irrelevant variables, 14 influential variables are selected, and the prediction performance of different models is evaluated.

**Table 4:** The prediction evaluation results.

Algorithms	MSE	MAPE	*R* ^2^
OLS	0.00148	0.55279	0.93711
Regression tree	0.00235	0.67984	0.90022
Bagging	0.00061	0.23424	0.97418
Random forest	0.00092	0.32716	0.96112
Boosting	0.00061	0.24972	0.97403
Xgboost	0.00060	0.28470	0.97506
**Extratrees**	**0.00049**	**0.21164**	**0.98121**

Source: our elaborations.

Notes: To ensure prediction accuracy, all parameters of the six machine learning models are set to the ideal value when the learning rate is 0.1, and further adjustment of parameters is spared.

Table 4 shows three conclusions. First, all the five machine learning algorithms selected in this paper, except the RT, demonstrate stronger prediction performance in predicting carbon emissions than the traditional OLS model, which verifies the utilization of the machine learning algorithms. Second, although the results of these algorithms are close in value, the Extra-trees is superior by providing the smallest MSE and MAPE values and the largest R^2^. This suggests that the Extra-trees is the optimal machine learning algorithm for predicting carbon emissions in China.

### Variable importance

**Fig. 2:**
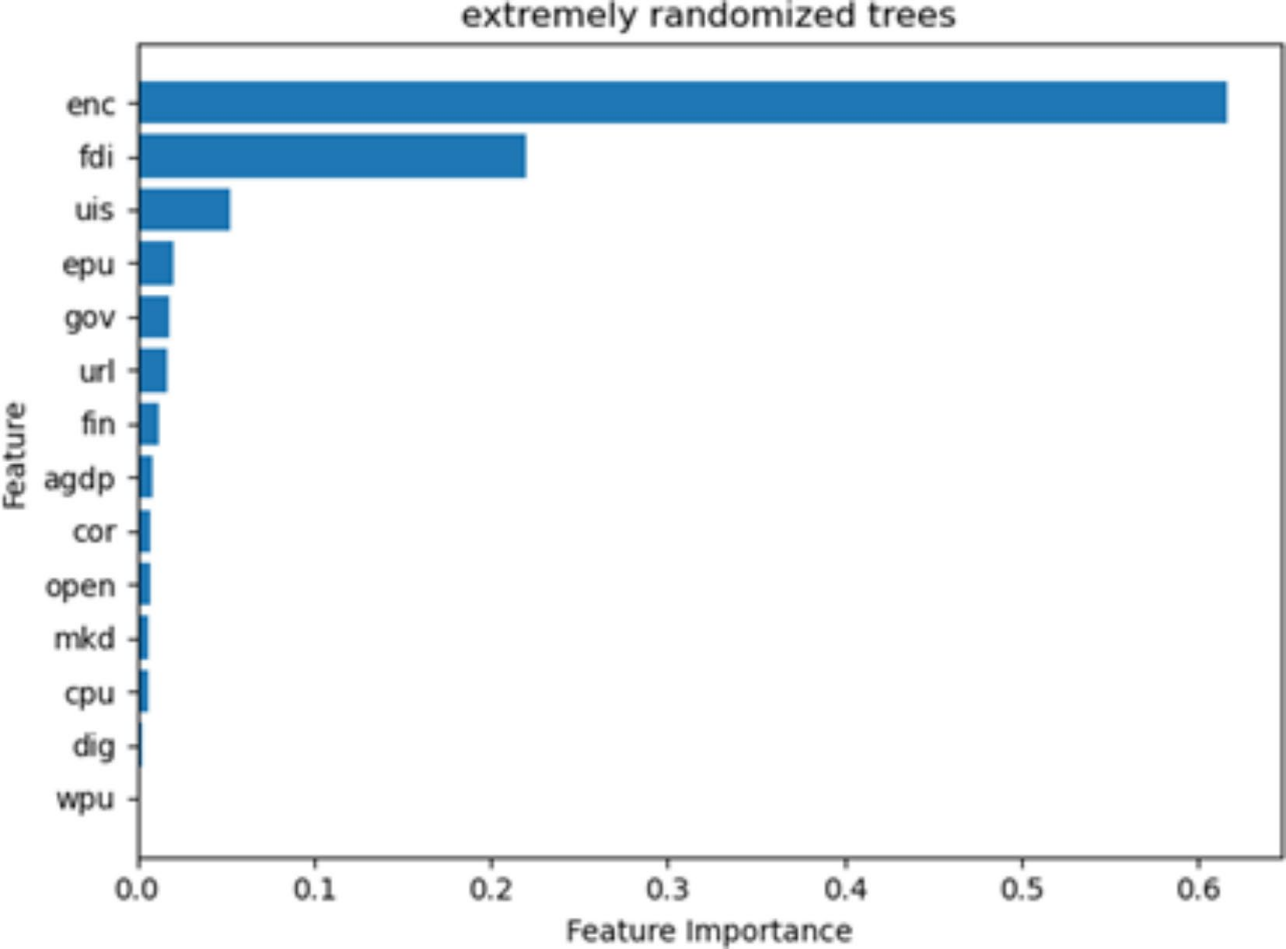
Variables importance ranking. Source: our elaborations.

The advantage that integrated machine learning models based on decision trees have is the ability to determine the order of importance of each influencing factor in predicting carbon emissions. The Extra-trees algorithm is carried out for variable importance analysis^57^, and Fig. 2 reports the importance ranking of 14 feature variables.

The study found that CE is still generated by internal production and economic activities, with energy consumption being the most influential internal factor. This result is shared by many scholars, as^58^ also argued that energy consumption is the main factor leading to CE, and that a rapid increase in energy use leads to a significant increase in carbon dioxide emissions. Next, the external economic factor of FDI is a secondary factor that affects carbon emissions and has received equal attention in carbon emissions research. Like the study of^59^, the main direction is to study whether the environmental effect of FDI is a pollution paradise or a pollution halo. Most importantly, uncertainty factors also seem to be a key factor affecting carbon emissions, especially the EPU, which suggests that EPU is also not negligible. Existing studies have examined the impact of uncertainty factors on China’s carbon emission efficiency less, and most of them still focus on internal economic and external economic factors.

### PDPs analysis

**Fig. 3:**
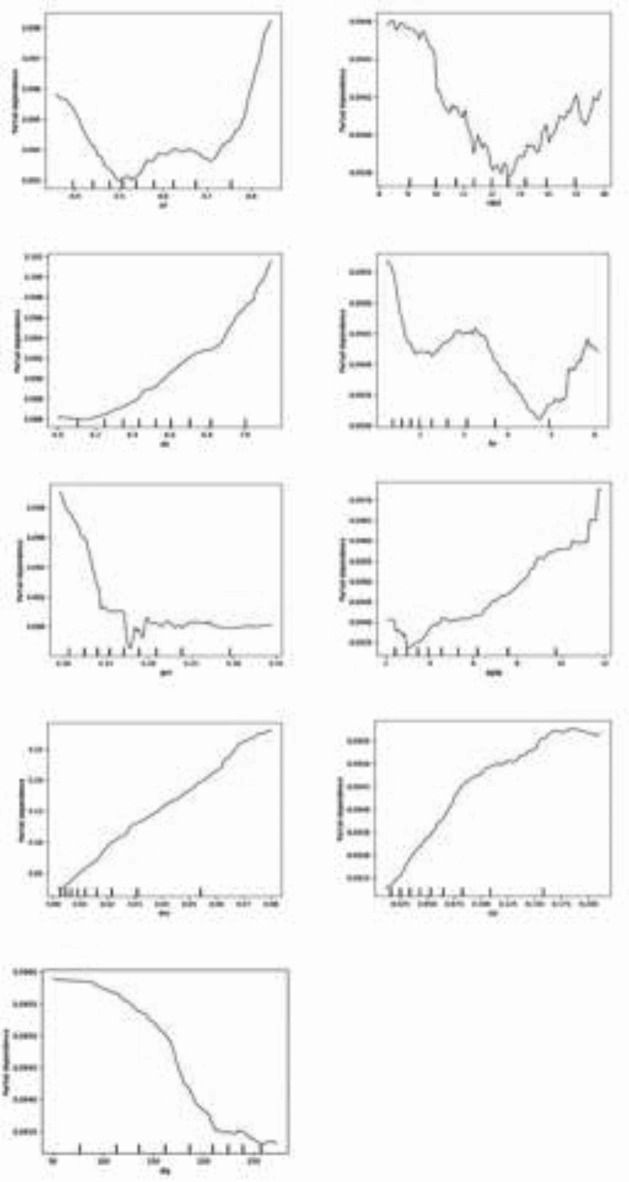
PDP analysis of domestic economic factors. Source: our elaborations. Notes: The vertical axis in each PDP indicates carbon emissions, and the horizontal axis shows the input variables.

**Fig. 4:**
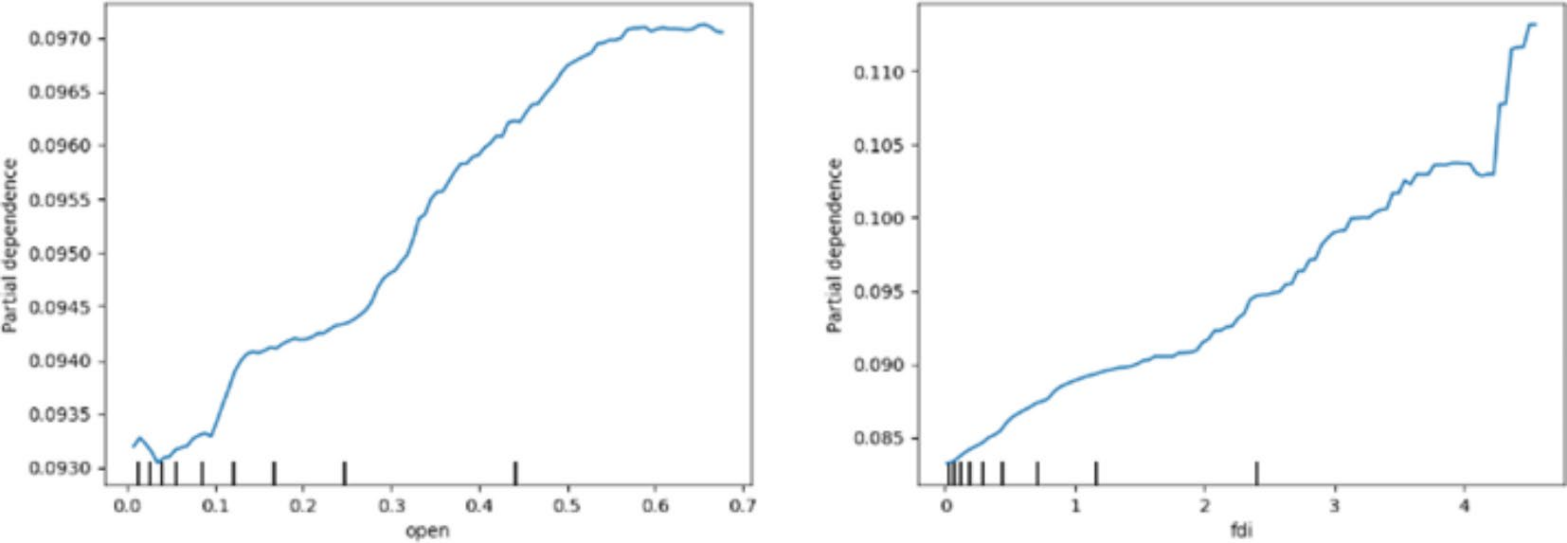
PDP analysis of external economic factors. Source: our elaborations.

**Fig. 5:**
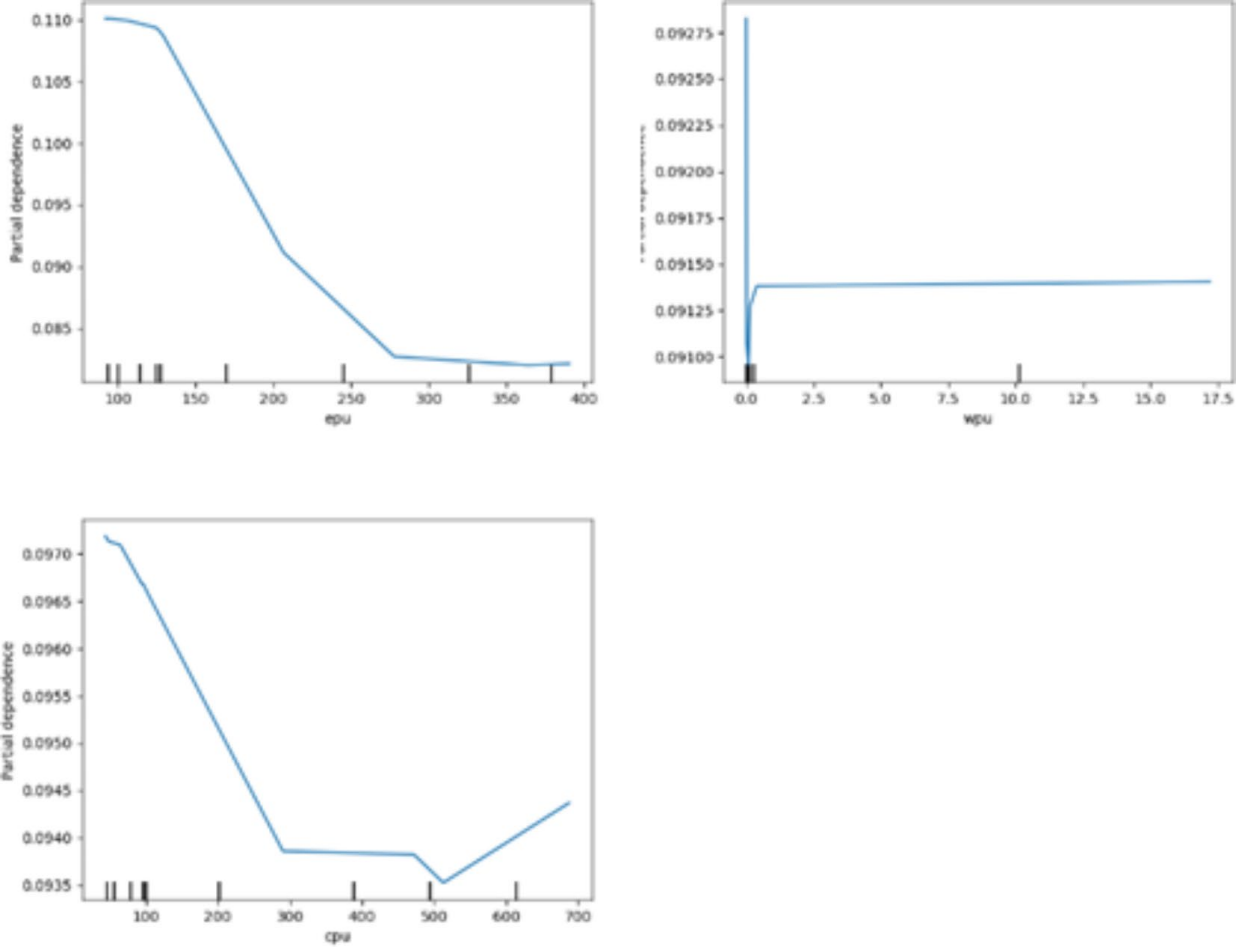
PDP analysis of policy uncertainty factors. Source: our elaborations.

The Extratrees algorithm not only allows for ranking the importance of variables but also enables the exploration of the nonparametric and quantile relationships, also known as PDPs analysis. Figures 3 and 4, and 5 visualize the marginal effects of domestic economic, external economic, and policy uncertainty factors on China’s carbon emissions, respectively.

Figure 3 presents the PDPs of nine domestic economic factors on carbon emissions. **First**, we find that URL, UIS, AGDP, ENC, and COR have a positive effect on CE, which is supported by the results of previous studies, such as^60^, who find that industrialization, economic growth, and energy consumption have a positive and significant effect on CE in Chin^61^. found that command environmental regulation does not significantly affect regional carbon neutrality. **Second**, as MKD rises, it tends to reduce and increase carbon emissions, with the most potent suppression when MKD is in the 6/10th percentile. For example^62^, finds that excessive marketization causes a lowering of the market entry barrier, leading to vicious competition among enterprises. **Third**, FIN seems to be less stable, although, in the lower quantiles, it suppresses the increase^63^. also find it. **Fourth**, GOV can effectively curb CE, as^23^ find that GOV is essential in reducing CE. **Fifth**, DIG significantly reduces carbon emissions in China, but the alleviating effect is not linear. Cities in the 2/10th to 5/10th deciles of the DIG have the most significant carbon mitigation effect^64^. also suggests that the carbon reduction effect is also weaker in cities where DIG is less developed.

Figure 4 shows the PDPs of the external economic factors. First, the increase of OPEN brings about a significant increase inCE. Although there are fewer observations in the tail region of OPEN and the results in the tail region may not be credible, it is still important to notice the strong effect of excessive openness to the outside world on pollution in a few cities. Second, the increase in FDI has a positive effect on CE, implying that FDI exacerbates the increase in CE. The rate of increase is more moderate in the lower quartile regions of FDI, but in the higher quartile regions of FDI, there is a near-vertical increase. Therefore, cities with high foreign direct investment need to the problem of large increases in CE. The results of this study also prove the “pollution paradise hypothesis”. That is, TNCs tend to transfer polluting industries or their production processes to developing countries for the sake of saving the cost of environmental management and other factors, thus aggravating environmental pollution in the host countries.

Figure 5 shows the partial dependence plot of the three major uncertainty factors, and the analysis of the estimated coefficients reveals that EPU has the greatest impact on CE, followed by CPU and WPU. First, EPU is the most among the three major uncertainty factors, which is also consistent with the findings of the variable importance ranking. The effect is not significant from 1/10th to 4/10th quantile of EPU, but by the 5/10th quantile, the increase in EPU significantly reduces carbon emissions until the 7/10th quantile when this negative effect gradually slows down. And after the 7/10th quantile, EPU is again insignificant. Second, WPU shows a sharp decline and then rebound to remain stable. At WPU quartiles 1/10 to 8/10, a “sudden stop” occurs. However, the phenomenon is short-lived, and at the 8/10 to 9/10 quantile of the WPU, carbon emissions increase rapidly to a certain level and remain stable. This suggests that WPU should be noticed as soon as it appears. Third, CPU and CE is a nonlinear one that decreases and then increases. At the 1/10th to 6/10th quartile of CPU, it decreases CE. Subsequently in the 6/10 to 8/10 quartile, CPU is insignificant. But in the tail region, CPU again increases carbon emissions.

### Research on urban heterogeneity

The size of a city depends on its population. The performance of different city types varies^65^. To examine the heterogeneous influence of the difference in urban population volume on carbon emissions in China, according to *the Notice on Adjusting the Criteria of Urban Size Classification released by the State Council* in 2014, we divide the cities into three categories (i.e., small-, medium- and large-sized cities) in accordance to their population, where 0.5 and 1 million are set as the boundaries.

**Fig. 6:**
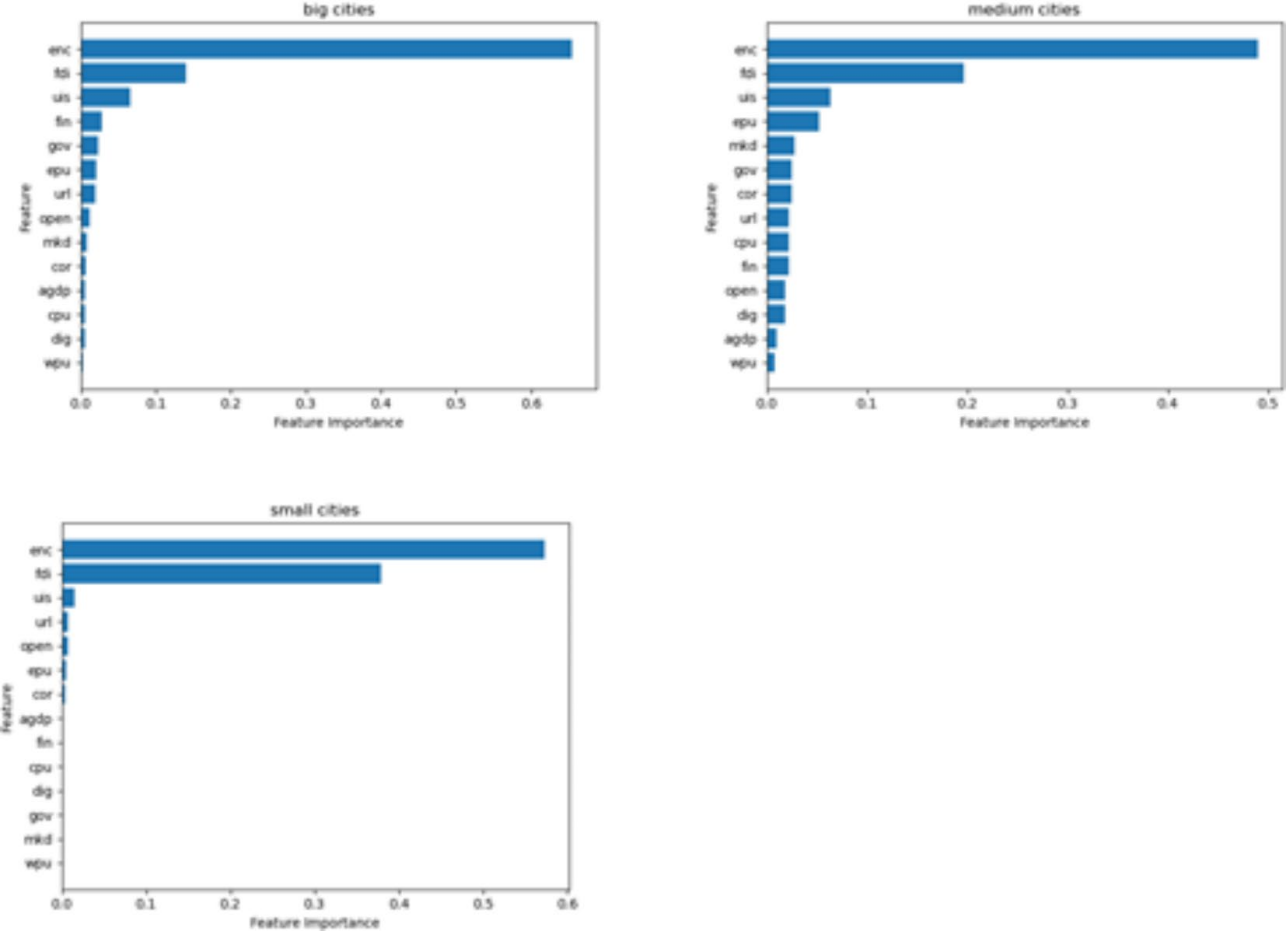
The heterogeneous results on city size. Source: our elaborations.

Figure 6 reports the results of the analysis of city size heterogeneity, and we find that the most important variable affecting carbon emissions is still ENC, regardless of small, medium and large cities. This implies that the reduction of traditional energy consumption is imminent. This requires China to increase its investment in energy transformation and upgrading. In addition, the extent of FDI’s impact manifests itself differently in different types of cities, but it will be greater in small and medium-sized cities. Polluting firms prefer medium-sized and small cities as pollution shelters, while large cities will pay more attention to FDI investment in R&D of cleaner production technologies and equipment. Finally, EPU is still the strongest among the uncertainty factors, but it has different performance in different sizes of cities, with EPU being more significant in small and medium-sized areas and weaker in large cities. On the one hand, this is inseparable from the fact that enterprises in large cities have a green development awareness and technological innovation. On the other hand, well-developed market information disclosure, social responsibility systems and strong environmental regulation are also major contributors to the ability of large cities to withstand the risk of uncertainty.

### Discussions

This experiment is conducted from three perspectives: internal, external and uncertainty. We find that the domestic economic factors of energy consumption (ENC) and industrial structure (UIS) are the most important influencing factors. Similar to the results of many studies, the high use of traditional energy sources remains the underlying cause, for example^58^. However, considering that China is increasingly affected by various external shocks as well as policy uncertainties in the context of the global economic downturn, the extent of their contribution to carbon emissions is interesting to research. Finally, cities of different sizes need to be customized in their choice of carbon reduction measures, so research on city-size heterogeneity is also necessary.

First, the external shocks. There is no clear conclusion in the existing research on whether the impact of FDI on the environment is the “Pollution Paradise Hypothesis” or the “Pollution Halo Hypothesis”. The experiments in this paper prove the Polluted Paradise Hypothesis. ^66^ and ^67^ argue that other countries favor FDI to shift the industry chain that generates high pollution to China, increasing carbon emissions through scale and structural effects. However, this is contrary to^68^, the environmental effect of FDI brings about a “pollution halo”. That is, the clean technology or standards adopted by TNCs will spread to local polluting enterprises, contributing to the improvement of our environment and emphasizing FDI’s technological effect.

Second, the policy uncertainty. (1) An increase in EPU significantly reduces carbon emissions. The reason is that in an environment of high EPU, enterprises and banks are afraid to take the risk of investing for fear that it will affect the return on investment. This results in a rapid deterioration of the economic development environment, enterprises face serious information deficiencies and business risks, significantly reducing the scale of production and investment activities. The eventual moratorium on the use of traditional energy sources is not conducive to productive activities^7^. concur with this view, which results from the cost effect of EPU. However, it deviates from the findings of^69^ who argued that EPU increases carbon emissions directly or indirectly through the investment channel. (2) Carbon emissions decrease rapidly at the onset of WPU, then rebound and remain stable. As WPU is accompanied by the spread of a pandemic and fluctuates over a short period of time, the emergence of a pandemic leads to a sudden cessation of economic activity and a sharp decrease in carbon emissions. However, this phenomenon lasts for a relatively short period of time, and as the pandemic is cured and economic activity resumes, carbon emissions quickly rebound and level off. However, in the tail region of the WPU, there are fewer observations, so the WPU should be noticed as soon as it occurs, otherwise it can have a significant impact^8^. also argued that the existence of the WUC would lead to a rapid reduction in carbon emissions due to the sudden stop in economic activity. (3) CPU and CE have a more complex relationship. At lower levels, with the increase in CPU, pushes the economy towards cleaner production, thus reducing fossil fuel consumption. However, this substitution effect is found to be absent in the high quantiles of CPU region as people shift to consume more fossil energy, thus rendering corporate green innovation less effective and ultimately leading to increased carbon emissions^70^. also share this view.

Finally, urban scale heterogeneity. (1) Foreign direct investment has a greater impact on small and medium-sized cities, probably because the “pollution paradise hypothesis” exists mainly in small and medium-sized cities with lax environmental enforcement measures. In the face of greater environmental pressures, the state prioritizes the optimization of the environment in large cities and reduces administrative resources for environmental enforcement in small and medium-sized cities. As a result, polluting firms find refuge in these areas, leading to a strong impact of FDI in small and medium-sized cities^71^. also share this view. (2) EPU has a more significant impact in small and medium-sized cities and a weaker impact in large cities. Large cities have a well-developed trade structure and strong confidence in residents’ consumption and business investment. EPU’s reduction in consumption through precautionary savings and wealth effects is not evident. Enterprises can also capture investment opportunities under uncertain conditions and mitigate risks, improving the ability of large cities to withstand uncertain risks. On the contrary, enterprises in small and medium-sized cities, whose awareness of green development responsibility and green innovation technology are not at a high level, market information disclosure and social responsibility system still need to be improved, environmental regulation still need to be strengthened, are more susceptible to the impact of EPU. Similarly^72^, argued that EPU on energy efficiency in different quartiles of urban distribution in China.

## Conclusions and policy recommendations

Many scholars have made great contributions to the field of carbon emission reduction and have identified many factors that affect carbon emissions in China. Among them, energy consumption has been identified as the root cause of China’s high carbon emissions. Of course, a few scholars also believe that certain external investment and policy uncertainty factors are also key factors affecting carbon emissions. However, existing studies have not uniformly included these influencing factors into the research framework for analysis. Therefore, this paper based on adaptive lasso and multiple integrated machine learning. The key factors affecting carbon emissions in 254 Chinese cities are identified from three aspects: internal economic factors, external economic factors and uncertainty factors. And the interpretability analysis of the prediction is carried out using variable importance and partial dependency graph. Cities are categorized into small, medium and large according to their size and analyzed for heterogeneity.

Similar to the results of most studies, e.g^27^. We find that energy consumption is indeed a fundamental factor influencing China’s high carbon emissions. Foreign direct investment (FDI) and economic policy uncertainty are important external factors influencing carbon emissions, and this influence is even higher than most internal factors. In addition, Extre-trees, an integrated machine learning model based on decision tree modeling, has the best performance in predicting urban carbon emissions. The model has unique advantages in terms of predictive interpretability, including variable importance ranking and partial dependency graph analysis. Our experiments affirm the contribution of traditional energy use. Furthermore, foreign direct investment (FDI) increases carbon emissions through scale and structural effects, proving the “pollution paradise” hypothesis of environmental effects. Economic policy uncertainty can exacerbate shrinkage of firms to reduce carbon emissions through the cost effect, which is accompanied by a decrease in economic activity and is detrimental to the country’s economic development. Finally, the heterogeneity analysis of city size reveals energy consumption as a common driver. Besides, large-sized cities should focus on the digital transformation of traditional finance, medium-sized cities on EPU, and small-sized cities on FDI.

We provide relevant policy recommendations. First, high consumption of traditional energy sources remains the root cause of high carbon emissions. Therefore, fully develop and utilize China’s extremely abundant wind, light, and water energy resources, accelerate the transition from traditional energy to renewable energy, realize the continuous substitution of black energy, and make simultaneous efforts from both the demand side and the supply side in order to dramatically reduce the demand for coal consumption^73^. also argue that renewable energy can alleviate the carbon emission problem in OECD. Second, China needs government departments and enterprises at all levels to introduce foreign investment of high quality and high efficiency in a selective and targeted manner. Taking foreign environmental protection requirements as the standard, introducing advanced technologies for green production and guiding foreign investment to expand into green research and development. Reducing the scale and structural effects and realizing the environmental effects of the FDI pollution halo by expanding the technological effects. Third, rising EPUs and unstable economic policies reduce the dynamism of economic development and make it difficult to achieve green and sustainable development. The Chinese government should endeavor to maintain the continuity and stability of domestic economic policies and prudently adjust economic policies. It should adopt a firmer policy stance to reduce economic policy volatility and accelerate the optimization of corporate energy consumption structure. Fourth, it identifies differences in influencing factors at different city scales and tailors its recommendations to the local context. In addition to direct measures to reduce energy use and accelerate energy green transformation, Large cities should fully utilize the emission reduction effect of digital finance and pay attention to its future development, The focus is on digital transformation, emphasizing the scenario application of emerging information technology and accelerating the deep integration of the two; Medium-sized cities should pay more attention to the ability to withstand the risk of economic policy uncertainty, enhance the flexibility of macroeconomic policies, strengthen the coordination and linkage of policies, moderately expand effective investment, improve the institutional mechanism to promote consumption, release the potential of domestic demand, and effectively respond to the uncertainty of economic policies with the great resilience and the certainty of sustained and healthy development; Smaller cities need to foreign direct investment on productive carbon emissions, take the “dual-carbon” goal as a guide, make digitalization, greening and intelligence high-quality requirements for the introduction of foreign direct investment, accelerating transformation, the green and low-carbon upgrading of foreign investment.

Some limitations will hopefully be addressed in future research. First, the experimental dataset is based on various factors studied in 254 cities in China. Then future research can go deeper into the enterprise level and consider experiments at the micro level, which can identify supply chain risks more precisely. Second, Second, the selection of optimal parameters determines the goodness of the predictive model, which requires parameter tuning using hyperparametric methods. So that machine learning models can be optimized from different tuning methods in future research. Finally, more sophisticated deep learning models may have better experimental results in predicting larger datasets compared to machine learning models, such as ANN and MARS models^25,74^. Therefore, experiments using neural network models could be considered in future research.

## Electronic supplementary material

Below is the link to the electronic supplementary material.

Supplementary Material 1

## Data Availability

The datasets used and analysed during the current study available from the corresponding author on reasonable request.

## Abbreviations

Extra-trees: Extremely Randomized Trees
MSE: Mean Square Error
MAPE: Mean Absolute Percentage Error
R2: Goodness of Fit
PDPs: Partial Dependence Plots
CE: Carbon emissions
URL: Urbanization rate
MKD: Marketability
UIS: Industrial structure
HCL: Human capital levels
FIN: Financial development
GOV: Government intervention
AGDP: Economic levels
ENC: Energy consumption
MAR: Market-inspired environmental regulation
COR: Command-based environmental regulation
GTFP: Green Total Factor Productivity
GTEC: Green Technology
DIG: Digital finance
FTE: Fintech Development
OPEN: Opening-up
FDI: Foreign Direct Investment
EPU: Economic policy uncertainty
WPU: World pandemic uncertainty
CPU: Climate policy uncertainty

## References

[1] Ipcc, S. R. Climate change 2022: Impacts, adaptation and vulnerability[EB/OL]. https://www.ipcc.ch/report/ar6/wg2/

[2] Tang, J. X., Gong, R. Z., Wang, H. L. & Liu, Y. X.Scenario analysis of transportation carbon emissions in China based on machine learning and deep neural network models. *Environ. Res. Lett. ***18** (6), 1–13. 10.1088/1748-9326/acd468 (2023).

[3] Tang, J. X., Gong, R. Z., Wang, H. L. & Liu, Y. X.Scenario analysis of transportation carbon emissions in China based on machine learning and deep neural network models. *Environ. Res. Lett. ***18** (6). 10.1088/1748-9326/acd468 (2023).

[4] Zhou, W. W., Cao, X. M., Dong, X. F. & Zhen, X. The effects of carbon-related news on carbon emissions and carbon transfer from a global perspective: evidence from an extended stirpat model. *J. Clean. Prod. ***425**10.1016/j.jclepro.2023.138974 (2023).

[5] Hao, L. N., Umar, M., Khan, Z. & Ali, W. Green growth and low carbon emission in g7 countries: how critical the network of environmental taxes, renewable energy and human capital is? *Sci. Total Environ. ***752** (1), 1–10. 10.1016/j.scitotenv.2020.141853 (2021). 10.1016/j.scitotenv.2020.141853 32889278

[6] Wang, Q. & Wang, S. S. Preventing carbon emission retaliatory rebound post-covid-19 requires expanding free trade and improving energy efficiency. *Sci. Total Environ. ***746** (1), 1–15. 10.1016/j.scitotenv.2020.141158 (2020). 10.1016/j.scitotenv.2020.141158 PMC7373025 32745860

[7] Lu, H. Z., Gao, Q. J. & Li, M. Does economic policy uncertainty outperform macroeconomic factor and financial market uncertainty in forecasting carbon emission price volatility? Evidence from China. *Appl. Econ. ***54**, 6427–6443. 10.1080/00036846.2022.2156470 (2022).

[8] Sikarwar, V. S., Reichert, A., Jeremias, M. & Manovic, V. Covid-19 pandemic and global carbon dioxide emissions: a first assessment. *Sci. Total Environ. ***794** (1), 1–7. 10.1016/j.scitotenv.2021.148770 (2021). 10.1016/j.scitotenv.2021.148770 PMC8441827 34225159

[9] Yilanci, V. & Ursavas U.Dynamic relationship between carbon emissions and climate policy uncertainty: a dynamic symmetric and asymmetric fourier causality analysis. *Environ. Eng. Manag. J. ***22** (1), 105–124. 10.30638/eemj.2023.010 (2023).

[10] Mirza, F. M. & Kanwal, A. E. Consumption, carbon emissions and economic growth in Pakistan: dynamic causality analysis. *Renew. Sustainable Energy Reviews*. **72**, 1233–1240. 10.1016/j.rser.2016.10.081 (2017).

[11] Xiao, Y. P. et al. D.Spatiotemporal differentiation of carbon emission efficiency and influencing factors: from the perspective of 136 countries. *Sci. Total Environ. ***879**10.1016/j.scitotenv.2023.163032 (2023). 10.1016/j.scitotenv.2023.163032 36965718

[12] Alessi, L. & Detken C.Identifying excessive credit growth and leverage. *J. Financial Stab. ***35**, 215–225. 10.1016/j.jfs.2017.06.005 (2018).

[13] Yu, W., Xia, L. & Cao Q.Forecasting digital economy of China using an adaptive lasso and grey model optimized by particle swarm optimization algorithm. *J. Intell. Fuzzy Syst. ***44** (2), 2543–2560. 10.3233/jifs-222520 (2023).

[14] Liu, W. D., Jiang, W. B., Tang, Z. P. & Han, M. Y.Pathways to peak carbon emissions in China by 2030: an analysis in relation to the economic growth rate. *Sci. China-Earth Sci. ***65** (6), 1057–1072. 10.1007/s11430-021-9901-y (2022).

[15] Zou, H. The adaptive lasso and its oracle properties. *J. Am. Stat. Assoc. ***101** (476), 1418–1429 (2006).

[16] Li, W., Zhang, S. & Lu C.Research on the driving factors and carbon emission reduction pathways of China’s iron and steel industry under the vision of carbon neutrality. *J. Clean. Prod. ***357** (1), 1–26. 10.1016/j.jclepro.2022.131990 (2022).

[17] Guo, X. M. & Fang, C. L.How does urbanization affect energy carbon emissions under the background of carbon neutrality? *J. Environ. Manage. ***327**10.1016/j.jenvman.2022.116878 (2023). 10.1016/j.jenvman.2022.116878 36470189

[18] Zhang, J., Wang, K. Q., Zhao, W. D., Han, Y. & Miao X.Corporate social responsibility and carbon emission intensity: is there a marketization threshold effect? *Emerg. Markets Finance Trade *. **58** (4), 952–964. 10.1080/1540496x.2020.1854219 (2022).

[19] Dong, B. et al. R.Carbon emissions, the industrial structure and economic growth: evidence from heterogeneous industries in China. *Environ. Pollut. ***262** (1), 1–12. 10.1016/j.envpol.2020.114322 (2020). 10.1016/j.envpol.2020.114322 32179222

[20] Lo Re, M., Veglianti, E., Parente, F., Monarca, U. & Magazzino C.Economic network dynamics: a structural analysis of the international connectivity of Chinese manufacturing firms. *J. Economic Stud. ***50** (8), 1585–1600. 10.1108/JES-10-2022-0531 (2023).

[21] Khan, M. Co(2)emissions and sustainable economic development: new evidence on the role of human capital. *Sustain. Dev. ***28** (5), 1279–1288. 10.1002/sd.2083 (2020).

[22] Kim, D. H., Wu, Y. C. & Lin, S. C.Carbon dioxide emissions and the finance curse. *Energy Econ. ***88**10.1016/j.eneco.2020.104788 (2020).

[23] Lin, B. Q. & Huang, C. C.Analysis of emission reduction effects of carbon trading: market mechanism or government intervention? *Sustainable Prod. Consum. ***33**, 28–37. 10.1016/j.spc.2022.06.016 (2022).

[24] Cai, C., Qiu, R. & Tu Y.Pulling off stable economic system adhering carbon emissions, urban development and sustainable development values. **10**: 1–13 doi: (2022). 10.3389/fpubh.2022.814656 10.3389/fpubh.2022.814656 PMC8866235 35223738

[25] Magazzino, C., Mele, M. & Schneider N.A new artificial neural networks algorithm to analyze the nexus among logistics performance, energy demand, and environmental degradation. *Struct. Change Econ. Dyn. ***60**, 315–328. 10.1016/j.strueco.2021.11.018 (2022).

[26] Kartal, M. T., Magazzino, C. & Pata, U. K.Marginal effect of electricity generation on co2 emissions: disaggregated level evidence from China by krls method and high-frequency daily data. *Energy Strategy Reviews*. **53**, 101382. 10.1016/j.esr.2024.101382 (2024).

[27] Magazzino, C. & Mele M.A new machine learning algorithm to explore the co2 emissions-energy use-economic growth trilemma. *Ann. Oper. Res. *10.1007/s10479-022-04787-0 (2022).

[28] Jiang, Q. C. & Ma, X. J.Spillovers of environmental regulation on carbon emissions network. *Technol. Forecast. Soc. Chang.* **169**10.1016/j.techfore.2021.120825 (2021).

[29] Yang, Y. Z., Wei, X. J., Wei, J. & Gao X.Industrial structure upgrading, green total factor productivity and carbon emissions. *Sustainability*. **14** (2). 10.3390/su14021009 (2022).

[30] Habiba, U., Xinbang, C. & Anwar A.Do green technology innovations, financial development, and renewable energy use help to curb carbon emissions? *Renew. Energy*. **193**, 1082–1093. 10.1016/j.renene.2022.05.084 (2022).

[31] Yuan, B. & Cao X.Do corporate social responsibility practices contribute to green innovation? The mediating role of green dynamic capability. *Technol. Soc. ***68**, 101868. 10.1016/j.techsoc.2022.101868 (2022).

[32] Wang, H. L. & Guo, J. G. Impacts of digital inclusive finance on co(2)emissions from a spatial perspective: evidence from 272 cities in China. *J. Clean. Prod. ***355**10.1016/j.jclepro.2022.131618 (2022).

[33] Lu, Y., Guo, J. X., Ahmad, M. & Zhang, H. T.Can sci-tech finance pilot policies reduce carbon emissions? Evidence from 252 cities in China. *Front. Environ. Sci.* **10**10.3389/fenvs.2022.933162 (2022).

[34] Karim, A. E., Albitar, K. & Elmarzouky M.A novel measure of corporate carbon emission disclosure, the effect of capital expenditures and corporate governance. *J. Environ. Manage. ***290** (1), 1–8. 10.1016/j.jenvman.2021.112581 (2021). 10.1016/j.jenvman.2021.112581 33866086

[35] Doğan, B., Driha, O. M., Lorente, B. & Shahzad D. U.The mitigating effects of economic complexity and renewable energy on carbon emissions in developed countries. **29**(1): 1–12 doi: (2021). 10.1002/sd.2125

[36] Sun, W. & Dong, H. Y.Measurement of provincial carbon emission efficiency and analysis of influencing factors in China. *Environ. Sci. Pollut. Res. *10.1007/s11356-022-25031-z (2022). 10.1007/s11356-022-25031-z PMC9798366 36580252

[37] Li, X. L., Yuan, S. Q., Yu, Y. & Jiang, T. Y.Analysis of China’s heavy industry energy-related co2 emissions and its influencing factors: an input-output perspective. *Environ. Sci. Pollut. Res. *10.1007/s11356-022-24495-3 (2022). 10.1007/s11356-022-24495-3 36502474

[38] Zhong, S., Goh, T. & Su, B. Patterns and drivers of embodied carbon intensity in international exports: the role of trade and environmental policies. *Energy Econ. ***114**10.1016/j.eneco.2022.106313 (2022).

[39] Doğan, B., Balsalobre-Lorente, D. & Nasir, M. A.European commitment to cop21 and the role of energy consumption, fdi, trade and economic complexity in sustaining economic growth. *J. Environ. Manage. ***273**, 111146. 10.1016/j.jenvman.2020.111146 (2020). 32771851 10.1016/j.jenvman.2020.111146

[40] Ashena, M. & Shahpari, G. P. Uncertainty, economic activity, and carbon emissions: a nonlinear autoregressive distributed lag approach. *Environ. Sci. Pollut. Res. ***29** (34), 52233–52247. 10.1007/s11356-022-19432-3 (2022). 10.1007/s11356-022-19432-3 35257351

[41] Chang, L., Chen, K. M., Saydaliev, H. B. & Faridi, M. Z.Asymmetric impact of pandemics-related uncertainty on co2 emissions: evidence from top-10 polluted countries. *Stoch. Env. Res. Risk Assess. ***36** (12), 4103–4117. 10.1007/s00477-022-02248-5 (2022). 35873500 10.1007/s00477-022-02248-5 PMC9288206

[42] Golub, A. A. et al. J.Escaping the climate policy uncertainty trap: options contracts for redd. *Clim. Policy*. **18** (10), 1227–1234. 10.1080/14693062.2017.1422478 (2018).

[43] Huang, Y. S., Shen, L. & Liu H.Grey relational analysis, principal component analysis and forecasting of carbon emissions based on long short-term memory in China. *J. Clean. Prod. ***209**, 415–423. 10.1016/j.jclepro.2018.10.128 (2019).

[44] Kong, F., Song, J. B. & Yang, Z. Z.A daily carbon emission prediction model combining two-stage feature selection and optimized extreme learning machine. *Environ. Sci. Pollut. Res. ***29** (58), 87983–87997. 10.1007/s11356-022-21277-9 (2022). 10.1007/s11356-022-21277-9 35821323

[45] Su, C. W., Pang, L. D., Tao, R., Shao, X. & Umar M.Renewable energy and technological innovation: which one is the winner in promoting net-zero emissions? *Technol. Forecast. Soc. Chang. ***182** (1), 1–11. 10.1016/j.techfore.2022.121798 (2022).

[46] Charles, A. & Darne O.Backcasting world trade growth using data reduction methods. *World Econ. ***1** (1), 1–23. 10.1111/twec.13274 (2022).

[47] Mardani, A., Liao, H. C., Nilashi, M., Alrasheedi, M. & Cavallaro F.A multi-stage method to predict carbon dioxide emissions using dimensionality reduction, clustering, and machine learning techniques. *J. Clean. Prod. ***275**10.1016/j.jclepro.2020.122942 (2020).

[48] Sun, W. & Huang, C. C.Predictions of carbon emission intensity based on factor analysis and an improved extreme learning machine from the perspective of carbon emission efficiency. *J. Clean. Prod. ***338**10.1016/j.jclepro.2022.130414 (2022).

[49] Li, S. S., Siu, Y. W. & Zhao, G. Q.Driving factors of co2 emissions: further study based on machine learning. *Front. Environ. Sci. ***9**10.3389/fenvs.2021.721517 (2021).

[50] Yang, W. Y. & Zhou, S. H.Using decision tree analysis to identify the determinants of residents’ co < sub > 2 emissions from different types of trips: A case study of guangzhou, china. *J. Clean. Prod. ***277**10.1016/j.jclepro.2020.124071 (2020).

[51] Bai, F. A machine learning approach for carbon di oxide and other emissions characteristics prediction in a low carbon biofuel-hydrogen dual fuel engine. *Fuel*. **341**10.1016/j.fuel.2023.127578 (2023).

[52] Li, W., Zhang, S. H. & Lu C.Research on the driving factors and carbon emission reduction pathways of china?S iron and steel industry under the vision of carbon neutrality. *J. Clean. Prod. ***361**10.1016/j.jclepro.2022.132237 (2022).

[53] Ozgur, O., Karagol, E. T. & Ozbugday, F. C. J. 金.Machine learning approach to drivers of bank lending: evidence from an emerging economy. **7**(1): 29 (2021).

[54] Hoxha, J., Çodur, M. Y., Mustafaraj, E., Kanj, H. & El Masri A.Prediction of transportation energy demand in türkiye using stacking ensemble models: methodology and comparative analysis. *Appl. Energy*. **350**10.1016/j.apenergy.2023.121765 (2023).

[55] Li, T. S. et al. Q.Contributions of various driving factors to air pollution events: interpretability analysis from machine learning perspective. *Environ. Int. ***173**10.1016/j.envint.2023.107861 (2023). 10.1016/j.envint.2023.107861 36898175

[56] Owusu, S. M., Fu, C. B. & Hu Q.Examining economic policy uncertainty’s impact on environmental sustainability: Insights from nordic nations. *J. Clean. Prod. ***449**10.1016/j.jclepro.2024.141688 (2024).

[57] Shetewy, N., Shahin, A. I., Omri, A. & Dai, K. Z.Impact of financial development and internet use on export growth: new evidence from machine learning models. *Res. Int. Bus. Finance*. **61** (1), 1–12. 10.1016/j.ribaf.2022.101643 (2022).

[58] Zhong, W. Y. et al. Y.Accurate and efficient daily carbon emission forecasting based on improved arima. *Appl. Energy*. **376**10.1016/j.apenergy.2024.124232 (2024).

[59] Massimiliano, C., Cooray, A., Kuziboev, B. & Liu J.Chinese fdi outflows and host country environment. *J. Environ. Manage. ***366**10.1016/j.jenvman.2024.121675 (2024). 10.1016/j.jenvman.2024.121675 38971068

[60] Khan, K. & Su, C. W. Urbanization and carbon emissions: a panel threshold analysis. *Environ. Sci. Pollut. Res. ***28** (20), 26073–26081. 10.1007/s11356-021-12443-6 (2021). 10.1007/s11356-021-12443-6 33481196

[61] Yang, Y. H., Peng, Z. W. & Tang, D. L.The impact of heterogeneous environmental regulations on carbon neutrality in China: new evidence based on the spatial measurement model. *Energy Environ. *10.1177/0958305x221140578 (2022).

[62] Zhang, D. Y. .Marketization, environmental regulation, and eco-friendly productivity: a malmquist-luenberger index for pollution emissions of large Chinese firms. *J. Asian Econ. ***76**10.1016/j.asieco.2021.101342 (2021).

[63] Amin, A., Dogan, E. & Khan Z.The impacts of different proxies for financialization on carbon emissions in top-ten emitter countries. *Sci. Total Environ. ***740**10.1016/j.scitotenv.2020.140127 (2020). 10.1016/j.scitotenv.2020.140127 32927547

[64] Zhang, M. L. & Liu Y.Influence of digital finance and green technology innovation on China’s carbon emission efficiency: empirical analysis based on spatial metrology. *Sci. Total Environ. ***838** (1), 1–15. 10.1016/j.scitotenv.2022.156463 (2022). 10.1016/j.scitotenv.2022.156463 35660603

[65] Xu, L., Fan, M. T., Yang, L. L. & Shao, S. Heterogeneous green innovations and carbon emission performance: Evidence at china’s city level. *Energy Econ. ***99**10.1016/j.eneco.2021.105269 (2021).

[66] Singhania, M. & Saini N.Demystifying pollution haven hypothesis: role of fdi. *J. Bus. Res. ***123**, 516–528. 10.1016/j.jbusres.2020.10.007 (2021). 33100429 10.1016/j.jbusres.2020.10.007 PMC7572317

[67] Dogan, B., Balsalobre-Lorente, D. & Nasir, M. A.European commitment to cop21 and the role of energy consumption, fdi, trade and economic complexity in sustaining economic growth. *J. Environ. Manage. ***273**10.1016/j.jenvman.2020.111146 (2020). 10.1016/j.jenvman.2020.111146 32771851

[68] Khan, S., Yuan, H. P., Yahong, W. & Ahmad, F. Environmental implications of technology-driven energy deficit and urbanization: insights from the environmental kuznets and pollution hypothesis. *Environ. Technol. Innov. ***34**10.1016/j.eti.2024.103554 (2024).

[69] Mushtaq, M. et al. S.How does economic policy uncertainty impact co2 emissions? Investigating investment’s role across 22 economies (1997–2021). *Energy Rep. ***11**, 5083–5091. 10.1016/j.egyr.2024.04.069 (2024).

[70] Shang, Y. F., Han, D., Gozgor, G., Mahalik, M. K. & Sahoo, B. K.The impact of climate policy uncertainty on renewable and non-renewable energy demand in the United States. *Renew. Energy*. **197**, 654–667. 10.1016/j.renene.2022.07.159 (2022).

[71] Zhang, C., Tao, R., Yue, Z. H. & Su, F. B.Regional competition, rural pollution haven and environmental injustice in China. *Ecol. Econ. ***204**10.1016/j.ecolecon.2022.107669 (2023).

[72] Wei, W., Hu, H. Q. & Chang, C. P.Why the same degree of economic policy uncertainty can produce different outcomes in energy efficiency? New evidence from China. *Struct. Change Econ. Dyn. ***60**, 467–481. 10.1016/j.strueco.2022.01.001 (2022).

[73] Dogan, B., Driha, O. M., Lorente, D. B. & Shahzad U.The mitigating effects of economic complexity and renewable energy on carbon emissions in developed countries. *Sustain. Dev. ***29** (1), 1–12. 10.1002/sd.2125 (2021).

[74] Cartwright, H. M.Artificial neural networks, (3rd edn.)[M].in (eds),In the methods in molecular biology series,New York:Springer,2021.

