# Supplementary material for: A machine learning algorithm to explore the drivers of carbon emissions in Chinese cities
Source: Sci Rep. 2024 Oct 9;14:23609. doi: 10.1038/s41598-024-75753-y (PMC11464641; doi:10.1038/s41598-024-75753-y)
Supplement: Supplementary file 1 — Supplementary Material 1 [file 41598_2024_75753_MOESM1_ESM.docx]

**Appendix 1 Variable Definitions and Data Sources**

**Supplementary Table 1 Variable Definitions and Data Sources**

| Group | Variable Title | Unit | Description | Source | Website |
| --- | --- | --- | --- | --- | --- |
| Dependent  Variable | Carbon emissions  (CE) | billion tonnes | The total carbon emissions of each city are obtained by adding up the carbon emissions from electricity, gas and LPG, transport, and thermal energy consumption. | *China Urban Construction Statistical Yearbook*, *China Statistical Yearbook, China Urban Statistical Yearbook* | *http://www.tjcn.org/* |
| Domestic economic factors | Urbanization rate  (URL) | % | Urban Resident Population / Citywide Resident Population | China Economic Network | https://db.cei.cn/jsps/Home |
|  | Degree of marketability  (MKD) | Index | A system of indices in the form of indices measures the relative marketization process of municipalities directly under the Central Government of China. | China Economic Network | https://db.cei.cn/jsps/Home |
|  | Industrial structure  (UIS) | Index | Tertiary sector output/total GDP | China Economic Network | https://db.cei.cn/jsps/Home |
|  | Human capital levels  (HCL) | % | Number of general undergraduate and above population / City's resident population | China Economic Network | https://db.cei.cn/jsps/Home |
|  | Level of financial development  (FIN) | % | Year-end financial institution loan balance/total GDP | Wind | https://www.wind.com.cn/ |
|  | Level of government intervention  (GOV) | % | Local budget expenditure (million yuan) * 0.0001/GDP (billion yuan) | Wind | https://www.wind.com.cn/ |
|  | Economic development  (AGDP) | million | GDP per capita | China Economic Network | https://db.cei.cn/jsps/Home |
|  | Total energy consumption  (ENC) | billion tonnes of standard coal | Data on consumption of all types of fossil energy, including coal, coke, gasoline, paraffin, diesel, fuel oil, and natural gas. | Energy Statistics Yearbook for China and Cities | https://www.stats.gov.cn/ |
|  | Market-inspired environmental regulation  (MAR) | % | Word frequency count for environmental vocabulary/Government Work Report Word Frequency | 2011-2017: sewage charges  2018-2020: Environmental taxes | https://www.resset.cn/ |
|  | Command-based environmental regulation  (COR) | Index | Calculation of a composite index of urban pollutant emissions to characterize the intensity of the forced urban environment psychological adjustment | Energy Statistics Yearbook for China and Cities | https://www.stats.gov.cn/ |
|  | Green Total Factor Productivity  (GTFP) | Index | Measure urban total factor productivity growth by integrating an over-efficient SBM model that considers non-desired outputs and the Malmquist productivity index within a globally referenced data envelopment analysis framework. | China Economic Network | https://db.cei.cn/jsps/Home |
|  | Green Technology Innovation  (GTEC) | individual | Number of green invention patent applications | National Intellectual Property Database | https://www.cnipa.gov.cn/col/col61/index.html |
|  | Level of development of digital finance  (DIG) | Index | *Digital Financial Inclusion Index* compiled by Peking University Internet Finance Research Centre[[1]](#footnote-1) | Centre for Internet Finance Research, Peking University | https://idf.pku.edu.cn/docs/20210421101507614920.pdf |
|  | Level of Fintech Development  (FTE) | Index | Baidu News Keyword Frequency | Baidu News | https://news.baidu.com/ |
| External economic factors | Level of openness to the outside world  (OPEN) | % | Total import/export trade/GDP | China Economic Network | https://db.cei.cn/jsps/Home |
|  | Green Technology Innovation  (GTEC) | individual | Number of green invention patent applications | National Intellectual Property Database | https://www.cnipa.gov.cn/col/col61/index |
| Uncertainty factors | Economic policy uncertainty  (EPU) | Index | China Economic Policy Uncertainty Index | Huang and Luk (2020)^[1]^ | |
|  | World pandemic uncertainty  (WPU) | Index | World Pandemic Uncertainty Index | Ahir et al. (2019)^[2]^ | |
|  | Climate policy uncertainty  (CPU) | Index | Climate policy uncertainty | Ren et al. (2023)^[3]^ | |

**Appendix 2** **Traditional OLS regression and machine learning models**

(1) Linear regression (OLS)

Traditional OLS focuses on determining the interdependencies between variables and takes the functional form of equation (2.1):

$h_{\omega,b}(x)=\sum_{i=1}^{n} \omega_{i}x_{i}+b_{i}=\omega^{T}x+b$ (2.1)

Where and are the model parameters, i.e., the regression coefficients.

If the actual model is linear, then traditional OLS may have better regression results; however, the real world is always complex, and therefore machine learning using non-linear, non-monotonic response functions is more advantageous.

(2) Regression tree

A decision tree is a regression tree when applied to a regression problem. The criterion for splitting a node is "Minimum the sum of squares due to error." it is preferable that the residual sum of squares falls the most after the split, i.e., the sum of the residual sum of squares of the two child nodes is the smallest. To avoid overfitting, we need to use the penalty term for pruning, i.e., minimizing the objective function (2.2):

$\min_{T} \sum_{m=1}^{\left|T\right|} \sum_{x_{i}\in R_{m}} (y_{i}-\hat{y}_{R_{m}})^{2}+\lambda\cdot\left|T\right|$ (2.2)

Where$R_{m}$ is the endpoint of, and$\hat{y}_{R_{m}}$is the predicted value of that endpoint (the sample mean of this endpoint). $\sum_{x_{i}\in R_{m}} (y_{i}-\hat{y}_{R_{m}})^{2}$is the sum of squares due to error at the$m$endpoint and the cost$R(T)$is the summation over all endpoints$m=1,2,\cdots,\left|T\right|$. However, the regression tree algorithm is the weak learner most commonly used for integration learning, so given the data and the algorithm, its estimate for supervised learning is mainly deterministic.

(3) Bagging method (Bagging)

Bagging is the averaging of many regression trees, which can reduce the variance of the estimates and turn weak learners into strong learners by combining them, thus improving the prediction accuracy of the model. Assuming that the random variable$\left\{z_{1},\cdots,z_{n}\right\}$ is independently and identically distributed and the variance is$\sigma^{2}$, the variance of the sample mean$\bar{z}=\frac{1}{n}\sum_{i=1}^{n} z_{i}$ can be reduced by a factor of n, see equation (2.3):

$Var(\bar{z})=Var(\frac{1}{n}\sum_{i=1}^{n} z_{i})=\frac{1}{n^{2}}\sum_{i=1}^{n} Var(z_{i})=\frac{\sigma^{2}}{n}$ (2.3)

Where the covariances in equation (4) are all zero because$\left\{z_{1},\cdots,z_{n}\right\}$ are independent of each other, and $Var(z_{i})=\sigma^{2}$ (identically distributed). In addition, as the bagging method does not prune and allows the decision trees to grow as much as they like, this can reduce the bias of each decision tree and control then the variance.

(4) Random forest

Random forests are a particular case of bagging, which builds on bagging by randomly selecting only some of the variables as candidate splitting variables at each node of the regression tree when it is divided. Assuming there are p feature variables, selecting m variables randomly at one node as candidate splitting variables (the remaining (p-m) variables are not used). In contrast, m variables are again randomly selected (possibly differently) at the next node. And so on, and then do so for each decision tree in the random forest. For regression trees, it is generally recommended that$m=p/3$ variables are selected at random. This practice, called random feature selection, is intended to reduce the correlation between decision trees.

At each decision tree node, the random forest uses only a small number of variables. This method discards most variables and does not use all the information, increasing the bias. However, as different nodes are forced to split using other variables, the correlation between different decision trees is reduced, thus reducing the variance. Thus, in the trade-off between bias and conflict, the random forest trades off a small amount of preference for a more significant reduction in friction and, therefore, a reduction in mean squared error or total error.

(5) Lifting Tree (GBM)

A boosted tree means that each decision tree serves a different purpose, and users cannot change the relative positions between these sequentially planted decision trees at will. For boosting regression problems, the basis function can be estimated non-parametrically and approximated in the "function space" using "gradient descent." For the training data $\left\{x_{i},y_{i}\right\}_{i=1}^{n}$, if y is predicted as a function of$F(x)$, the expected loss function in the aggregate is $E_{y,x}L\left[y,F(x)\right]$. Therefore, in the "function space"$F(x)$, we find the optimal function$F^{*}(x)$to minimize the expected loss function, according to equation (2.4):

$F^{*}(x)\equiv\mathop{\mathrm{argmin}}\limits_{F} E_{y,x}L\left[y,F(x)\right]$ (2.4)

Where$L\left[y,F(x)\right]$ is the given loss function, and optimization is performed for the function space of $F(\cdot)$.

(6) XGBoost

If the sample size is large or for extensive data, the Python module XGBoost developed by Chen and Guestrin (2016) is more suitable. This method has two main advantages: first, XGBoost constructs the loss function, introduces regularization, and performs a second-order Taylor expansion. Linear regression can also be used as a base learner. It achieves this by constructing multiple base classifiers to minimize the objective function, with each step going to find the best classifier for the above regularity-constrained loss function. The second is a parallel algorithm that can improve efficiency. The gradient boosting method is still used, but many improvements have been made to the specific algorithm, including the use of Newton's method to calculate the direction of descent, the progress of the decision tree algorithm, the use of sparse matrix, etc., making the speed of computing increase significantly. The objective function is Eq. (2.5):

$Obj^{(t)}=\sum_{i=1}^{n} l(y_{i},\hat{y}_{i}^{(t-1)}+f_{t}(x_{i}))+\Omega(f_{t})=cons\tan t$ (2.5)

(7) Extremely randomized trees algorithm (Extremely randomized trees algorithm, abbreviated as extra-trees)

Extra-Trees are similar to the Random Forest algorithm, consisting of many decision trees. The main differences between Extreme-Trees and Random Forest are: firstly, Random Forest applies a Bagging model, Extra-Trees uses all the samples, only the features are randomly selected, and because the splitting is random, it gives better results than Random Forest. Secondly, a Random Forest gets the best splitting attributes within a random subset, while Extra-Trees get the splitting values completely randomly, thus enabling the splitting of the decision tree.

1. https://idf.pku.edu.cn/docs/20210421101507614920.pdf [↑](#footnote-ref-1)
